# A Target-Displaced Aptamer–cDNA Duplex Strategy on ERGO for Ultrasensitive Turn-On Electrochemical Detection of Ochratoxin A

**DOI:** 10.3390/s26061937

**Published:** 2026-03-19

**Authors:** Intan Gita Lestari, Seung Joo Jang, Tae Hyun Kim

**Affiliations:** Department of Chemistry, Soonchunhyang University, Asan 31538, Republic of Korea; intangita@sch.ac.kr (I.G.L.);

**Keywords:** Ochratoxin A (OTA), turn-on electrochemical aptasensor, aptamer–cDNA duplex displacement, G-quadruplex formation

## Abstract

**Highlights:**

**What are the main findings?**

- A turn-on electrochemical aptasensor was developed using a target-induced displacement of an aptamer–cDNA duplex on an ERGO-modified electrode.
- OTA binding triggers G-quadruplex formation of the aptamer, leading to accelerated electron transfer and a pronounced signal-on response.

**What are the implications of the main findings?**

- The duplex displacement and G-quadruplex folding strategy enables highly sensitive and low-background electrochemical detection of OTA.
- The ERGO-based, linker-free platform provides an electron-transfer catalyst and large surface area, leading to ultrasensitive detection of OTA.

**Abstract:**

Ochratoxin A (OTA) is a highly toxic mycotoxin commonly detected in food and agricultural products, requiring sensitive analytical methods for reliable monitoring. Herein, we report an ultrasensitive turn-on electrochemical aptasensor for OTA detection based on a target-induced displacement of an aptamer–complementary DNA (cDNA) duplex assembled on an electrochemically reduced graphene oxide (ERGO)-modified glassy carbon electrode (GCE). In the absence of OTA, a methylene blue (MB)-labeled aptamer hybridized with cDNA is immobilized on the ERGO surface via π–π stacking interactions, forming a rigid duplex that suppresses electron transfer and yields a low electrochemical signal. Upon OTA binding, the aptamer undergoes a conformational transition into a G-quadruplex structure, leading to dissociation of the cDNA strand. This target-induced folding brings the MB redox tag into close proximity to the ERGO surface, markedly accelerating electron transfer and enhancing the cathodic reduction current of MB, thereby producing a pronounced signal-on response in square-wave voltammetry (SWV). The ERGO-modified electrode provides a conductive and stable interface without chemical linkers. Under optimized conditions, the aptasensor shows a linear response to OTA from 10 fM to 100 pM with an ultralow LOD of 0.67 fM, together with high selectivity, good reproducibility, and satisfactory stability. This work demonstrates a simple and effective turn-on aptasensing strategy for sensitive electrochemical detection of OTA.

## 1. Introduction

**Figure 1:**
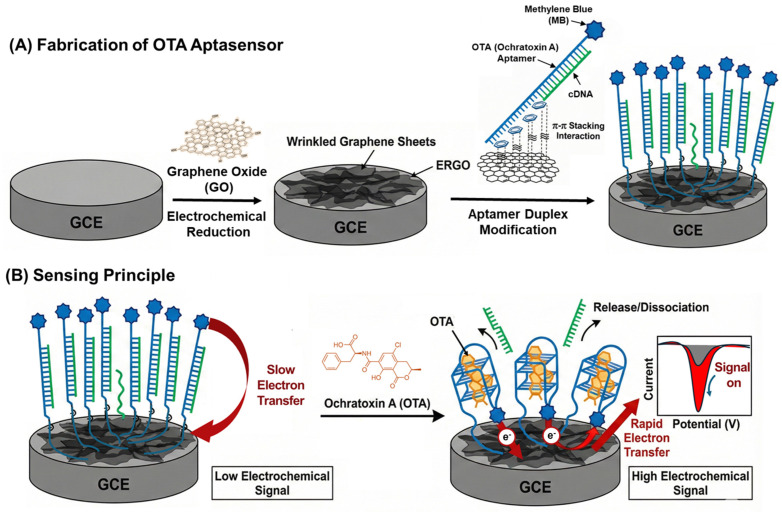
Schematic diagram of fabrication of OTA aptasensor and its sensing principle.

Ochratoxin A (OTA) is a hazardous mycotoxin produced by several Aspergillus and Penicillium species [1] and is frequently encountered in a wide range of food and agricultural commodities, including cereals [2], coffee [3], dried fruits [4], spices [5], and wine [6]. Because OTA can persist through food processing stages and has been linked to severe health risks, including nephrotoxicity [7] and carcinogenicity [8], sensitive and reliable monitoring methods are imperative for safeguarding public health and ensuring regulatory compliance. Conventional analytical techniques, such as high-performance liquid chromatography (HPLC) coupled with fluorescence detection [9] and liquid chromatography–mass spectrometry (LC–MS) [10], provide high sensitivity and excellent selectivity. However, these methods typically require costly instrumentation [11], skilled personnel [12], labor-intensive sample preparation [13], and centralized laboratory facilities [14], which limit their suitability for rapid screening [15] and on-site applications [16]. Consequently, there is increasing demand for portable, cost-effective, and user-friendly sensing platforms that enable trace-level detection of OTA in complex matrices. Electrochemical biosensors have emerged as attractive alternatives due to their intrinsic sensitivity [17], low power consumption [18], compatibility with miniaturized devices [19], and straightforward signal readout [20]. Among available recognition elements, aptamers are single-stranded DNA or RNA sequences generated through systematic evolution of ligands by exponential enrichment (SELEX) [21], and they offer distinct advantages over antibodies, including chemical stability [22,23], facile modification [24], reproducible synthesis [25], and reduced batch-to-batch variation [26]. These properties make aptamers particularly suitable for robust and scalable electrochemical sensing systems. Although electrochemical aptasensors have been widely explored for OTA detection, many reported designs rely on “signal-off” transduction [27,28], where target binding suppresses the redox response. Signal-off modes can suffer from relatively small dynamic signal changes and a higher susceptibility to matrix-induced false positives [29,30] (e.g., electrode fouling), which collectively reduce reliability in real samples. In contrast, “signal-on” strategies that generate an increased response upon target recognition are preferred because they provide a clearer analytical readout and improved signal-to-noise ratios [31], especially at ultra-low analyte concentrations [32]. Target-induced strand displacement using an aptamer–complementary DNA (cDNA) duplex provides an effective route to engineer signal-on electrochemical aptasensors [33]. In this framework, the aptamer is pre-hybridized with a short cDNA to form a duplex that stabilizes a defined initial conformation and controls the spatial position of a redox reporter. When the target is introduced, the aptamer preferentially binds the analyte, triggering duplex dissociation and the rearrangement of the sensing layer. This molecular switching mechanism can be deliberately designed to modulate the distance between a redox tag and the electrode surface, thereby amplifying the electrochemical response. For OTA, aptamer binding is commonly accompanied by a pronounced conformational change into a G-quadruplex structure [34], which can be leveraged to drive the displacement of cDNA and reposition a redox label closer to the electrode to accelerate electron transfer. In parallel with recognition-layer engineering, the choice of the electrode interface is critical for achieving high sensitivity and robust performance. Graphene-based materials offer high electrical conductivity [35], strong π–π interactions with nucleic acids [36], and efficient charge transport [37]. In particular, electrochemically reduced graphene oxide (ERGO) provides an enabling stable immobilization by electrochemical reduction [38], enhanced charge transport [39], and large surface area [40] due to wrinkled ERGO morphology. It can provide abundant anchoring sites for DNA assemblies. Moreover, ERGO supports non-covalent immobilization through π–π stacking [41], reducing dependence on additional coupling chemistries [42] and simplifying fabrication [23]. Taken together, a duplex displacement mechanism integrated with an ERGO interface is expected to offer a practical and sensitive platform for turn-on OTA detection. Herein, we propose an ultrasensitive “turn-on” electrochemical aptasensor that couples an ERGO-modified glassy carbon electrode (GCE) with a methylene blue (MB)-labeled aptamer–cDNA duplex sensing layer, as illustrated in Figure 1. In the initial state, the rigid duplex configuration hinders electron transfer, resulting in a low background signal. Upon OTA binding, the aptamer folds into a G-quadruplex structure with higher target affinity [43,44], inducing cDNA dissociation and bringing the MB redox tag into closer proximity to the ERGO surface, thereby producing a pronounced signal-on response. We systematically characterize the electrode interface and stepwise assembly, optimize key parameters (duplex concentration, accumulation time, and target incubation time), and evaluate analytical performance using square-wave voltammetry (SWV), along with selectivity, reproducibility, and stability tests. The resulting sensing strategy provides a simple, linker-free, and mechanistically interpretable route for sensitive electrochemical OTA monitoring, highlighting the broader potential of ERGO-supported duplex displacement designs for food safety analysis.

## 2. Materials and Methods

### 2.1. Materials

The methylene blue-labeled DNA aptamer (MB-Apt), previously reported in the literature [1], together with its partially complementary strand (cDNA), was synthesized by Bioneer Corporation (Daejeon, Republic of Korea). The nucleotide sequences were as follows [1]: the base strand was 5′-AAAAAAAAAAGATCGGGTGTGGGTGGCGTAAAGGGAGCATCGGACA-Methylene Blue-3′, and the complementary DNA (cDNA) strand was 5′-CCGATGCTCTCCTTACGCCACCCACACCCG-3′. Graphite powder, potassium permanganate (KMnO_4_), hydrogen peroxide (H_2_O_2_), sodium nitrate (NaNO_3_), sulfuric acid (H_2_SO_4_), phosphate-buffered saline (PBS, pH 7.4, 0.01 M), K_3_Fe(CN)_6_, tris-buffered saline (TBS; 0.1 M Tris-HCl, pH 7.6), magnesium chloride hexahydrate (MgCl_2_ ∙ 6H_2_O), and potassium chloride (KCl) were purchased from Sigma-Aldrich (St. Louis, MO, USA). All reagents were of analytical grade and utilized without additional purification.

### 2.2. Instruments

Electrochemical measurements were performed with a CHI660D electrochemical workstation (CH Instruments, Austin, TX, USA; Z-202306208148 at the Research Support Center for Bio-Bigdata Analysis and Utilization of Biological Resources) using a conventional three-electrode configuration, consisting of a platinum wire as the counter electrode, an Ag/AgCl electrode as the reference electrode, and a bare or modified glassy carbon electrode (GCE, 3 mm diameter) as the working electrode. All experiments were conducted at ambient temperature. Ultrasonic treatment was carried out using a Sonics Vibracell VC 505 sonicator (Sonics & Materials Inc., Newton, CT, USA) for 1 h at an amplitude of 28%, with a pulse sequence of 1 s on and 3 s off.

### 2.3. Preparation and Hybridization of cDNA/MB-Apt

The DNA duplex probe was prepared by hybridizing the aptamer strand with its partially complementary DNA (cDNA) in TM buffer (10 mM Tris-HCl, 100 mM KCl, and 1 mM MgCl_2_·6H_2_O). Briefly, 980 µL of TM buffer was mixed with 10 µL of the aptamer solution and 10 µL of the cDNA solution to yield a final aptamer concentration of 1 µM. The mixture was heated at 55 °C for 10 min and then cooled to room temperature for 30 min to facilitate annealing and the formation of duplex DNA (dsDNA) between the MB-aptamer and cDNA strands. The resulting aptamer probe was stored at 4 °C for overnight prior to use and was used within 24 h.

### 2.4. Synthesis cDNA/MB-Apt/ERGO-GCE

The cDNA/MB-Apt/ERGO-modified glassy carbon electrode (GCE) was prepared as follows. First, the GCE was sequentially polished with alumina slurries of 1.0, 0.3, and 0.05 μm particle sizes, followed by ultrasonic cleaning in a mixture of deionized (DI) water and ethanol (1:1, *v*/*v*) for 10 min each to remove residual particles. Graphene oxide (GO) was synthesized using a modified Hummers’ method [45] and subsequently dispersed in 10 mM PBS buffer (pH 7.4) by ultrasonication for 1 h to obtain a homogeneous suspension. The ERGO film was then electrodeposited onto the GCE surface by cyclic voltammetry in a GO solution (0.3 mg/mL in 10 mM PBS) over a potential range of −0.8 to 1.5 V (vs. Ag/AgCl) at a scan rate of 10 mV/s for three cycles. After electrodeposition, the ERGO-modified GCE (ERGO–GCE) was incubated in a 1 μM cDNA/MB-Apt duplex solution prepared in TM buffer for 40 min under optimized conditions to allow immobilization of the DNA probe onto the ERGO surface via π–π stacking interactions. Finally, the resulting cDNA/MB-Apt/ERGO–GCE was rinsed thoroughly with DI water and dried at room temperature prior to further use.

## 3. Results

### 3.1. Characterization

**Figure 2:**
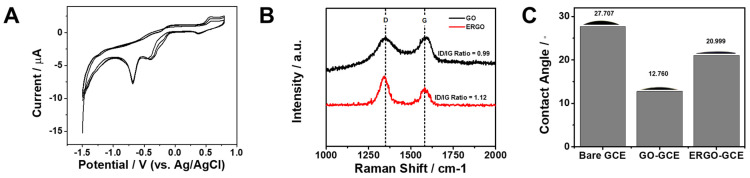
(**A**) Cyclic voltammograms recorded during the electrochemical reduction of GO to ERGO over three successive cycles, showing the progressive reduction behavior. (**B**) Raman spectra of GO and ERGO. The D band (~1350 cm^−1^) is associated with structural defects, while the G band (~1580 cm^−1^) corresponds to the graphitic structure of sp^2^ carbon. (**C**) Contact angle measurements of various modified electrodes, including bare GCE, GO-modified GCE, and ERGO-modified GCE.

The electrochemical reduction of graphene oxide (GO) to electrochemically reduced graphene oxide (ERGO) was carried out by cyclic voltammetry [45]. As shown in Figure 2A, the first cathodic scan exhibits a pronounced reduction peak in the range of approximately −0.6 to −0.8 V, which can be attributed to the electrochemical removal of oxygen-containing functional groups from the GO sheets. In subsequent cycles, the intensity of this reduction peak progressively decreased and eventually vanished, indicating the gradual depletion of electroactive oxygen functionalities and the effective conversion of GO into ERGO. Concomitantly, an overall increase in current response was observed after the reduction process, reflecting improved charge transfer characteristics and partial restoration of the sp^2^ carbon network [46]. These results demonstrate the successful in situ formation of a conductive ERGO layer on the GCE surface.

To further corroborate the structural transformation suggested by the electrochemical results, Raman spectroscopy was employed. As presented in Figure 2B, both GO and ERGO exhibit the characteristic D band (~1350 cm^−1^) and G band (~1580 cm^−1^), corresponding to the disorder-induced breathing mode of sp^2^ carbon rings and the in-plane stretching vibration of sp^2^ carbon atoms, respectively. Prior to peak analysis, the Raman spectra were baseline-corrected using OriginPro 9.0 (OriginLab Corporation, Northampton, MA, USA) to remove background fluorescence. After reduction, the intensity ratio of the D to G band (I_D_/I_G_) increased from 0.99 for GO to 1.12 for ERGO. This change suggests a modification in the defect structure accompanying deoxygenation, commonly associated with the formation of smaller but more numerous sp^2^ domains following removal of oxygen functionalities. Such structural rearrangement is consistent with the partial restoration of graphitic regions. The increased density of defect sites and edge-like structures is advantageous for electrochemical applications, as these features can provide additional active sites and facilitate interfacial electron transfer [47,48].

Since the removal of oxygen-containing functional groups not only alters the defect structure but also modifies the surface chemical composition, changes in surface wettability were further evaluated by contact angle measurements. As presented in Figure 2C, the bare GCE exhibited a contact angle of 27.7°, indicating a relatively moderate wettability. After GO deposition, the contact angle decreased markedly to 12.8°, consistent with the highly hydrophilic character of GO arising from its abundant oxygen-containing functional groups, such as hydroxyl, epoxy, and carboxyl moieties [49]. Following electrochemical reduction, the contact angle increased to 21.0° for the ERGO–GCE. This increase can be attributed to the partial removal of oxygen functionalities and the concomitant restoration of graphitic sp^2^ domains, which decreases surface polarity and lowers the surface free energy associated with polar interactions [50]. Although the wettability of ERGO remained higher than that of the bare GCE, the observed increase relative to GO reflects the successful deoxygenation process and structural rearrangement induced by electrochemical reduction [51,52]. These systematic variations in contact angles across the modification steps provide complementary evidence for the progressive transformation from GO to ERGO and support the successful in situ formation of the ERGO layer on the electrode surface.

To further verify the surface morphology changes associated with electrode modification, atomic force microscopy (AFM) analysis was performed. As shown in Figure S1A,B, the ERGO-modified GCE exhibited a characteristic wrinkled surface morphology, which is a typical structural feature of reduced graphene oxide layers. After immobilization of the cDNA/MB-Apt duplex on the ERGO-GCE, the surface morphology changed from a wrinkled structure to a more aggregated and irregular appearance (Figure S1C), indicating the successful assembly of the DNA sensing layer on the ERGO-modified electrode.

**Figure 3:**
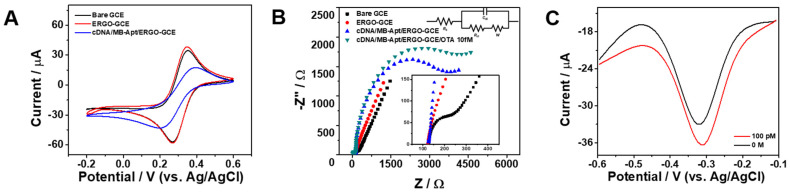
(**A**) CV curves of bare GCE, ERGO–GCE, and cDNA/MB-Apt/ERGO–GCE recorded in 0.1 M KNO_3_ containing 10 mM K_3_[Fe(CN)_6_] at a scan rate of 10 mV s^−1^. (**B**) Nyquist plots of the corresponding electrodes measured in the same electrolyte containing 10 mM K_3_[Fe(CN)_6_]. (**C**) SWV responses of cDNA/MB-Apt/ERGO–GCE before and after incubation with OTA (1 nM) in 10 mM Tris–HCl (pH 7.4).

To further investigate how these structural and surface changes influence the interfacial electrochemical properties, the modified electrodes were subsequently characterized by cyclic voltammetry (CV) and electrochemical impedance spectroscopy (EIS) using a standard redox probe solution containing 10 mM [Fe(CN)_6_]^3−^. Measurements were performed for the bare GCE, ERGO–GCE, and cDNA/MB-Apt/ERGO–GCE to monitor interfacial changes at each modification step. As shown in Figure 3A, the bare GCE displays a pair of well-defined and nearly reversible redox peaks with a peak-to-peak separation (ΔE_p_) of 0.083 V, characteristic of the [Fe(CN)_6_]^3−^. After ERGO deposition, ΔE_p_ slightly decreases to 0.080 V, accompanied by an increase in both anodic and cathodic peak currents. This behavior indicates improved electron transfer kinetics, which can be attributed to enhanced electroactive surface area provided by the ERGO layer. In contrast, upon immobilization of the cDNA/MB-Apt duplex onto the ERGO-modified electrode, ΔE_p_ increases markedly to 0.212 V, reflecting a slower electron transfer process. This change is reasonably ascribed to the formation of a negatively charged and relatively insulating DNA layer that partially blocks the electrode surface. The phosphate backbone of the DNA introduces electrostatic repulsion against the negatively charged [Fe(CN)_6_]^3−^ probe, thereby hindering its approach to the interface and increasing the resistance to charge transfer.

To further quantify the interfacial electron transfer characteristics and gain deeper insight into the kinetic changes induced by each modification step, electrochemical impedance spectroscopy (EIS) was subsequently performed. As shown in Figure 3B, the Nyquist plots display a high-frequency semicircle corresponding to the charge transfer resistance (R_ct_) and a low-frequency linear region associated with diffusion-controlled processes. The bare GCE exhibits a small semicircle with an R_ct_ value of approximately 0.05 kΩ, indicative of rapid electron transfer at the electrode–electrolyte interface. Following ERGO modification, the semicircle diameter decreases further and R_ct_ is reduced to ~0.02 kΩ, reflecting enhanced active surface area and improved charge transfer kinetics provided by the ERGO layer. After immobilization of the cDNA/MB-Apt duplex, a pronounced enlargement of the semicircle is observed, and equivalent circuit fitting yields an R_ct_ value of 2.65 kΩ. This increase indicates the formation of a kinetically resistive interfacial layer that impedes electron transfer between the electrode surface and the [Fe(CN)_6_]^3−^ redox probe. The elevated R_ct_ can be attributed to steric hindrance as well as electrostatic repulsion arising from the negatively charged phosphate backbone of the DNA layer, which restricts the approach of the negatively charged [Fe(CN)_6_]^3−^ species to the electrode interface. Upon OTA binding, the semicircle further enlarges (R_ct_ > 2.65 kΩ), suggesting additional interfacial impedance induced by target recognition. This behavior consists of conformational rearrangement of the aptamer and formation of a more compact interfacial structure, which further limits access of the redox probe to the electrode surface. Overall, the progressive increase in R_ct_ from bare GCE to ERGO–GCE and subsequently to cDNA/MB-Apt/ERGO–GCE provides strong evidence for the successful sequential modification of the electrode interface. Notably, the EIS trend reflects the interfacial blocking effect toward the negatively charged [Fe(CN)_6_]^3−^ probe, whereas the analytical square-wave voltammetry (SWV) signal originates from the MB tag whose electron transfer is enhanced by the reduced distance after target-induced folding. Having established the interfacial architecture and electron transfer characteristics of the modified electrode, the analytical signal response of the sensing platform was subsequently evaluated by SWV, as illustrated in Figure 3C. In the absence of OTA, the electrode exhibits a relatively low peak current corresponding to the baseline redox response of the methylene blue (MB)-labeled aptamer. Upon exposure to 1 nM OTA, a distinct increase in peak current is observed. This signal enhancement is attributed to target-induced structural rearrangement of the aptamer, which brings the MB redox reporter into closer proximity to the ERGO surface, thereby facilitating electron transfer. The clear difference in current response before and after OTA incubation verifies the effective implementation of the turn-on sensing mechanism and demonstrates the responsiveness of the engineered interfacial architecture.

### 3.2. Optimization

**Figure 4:**
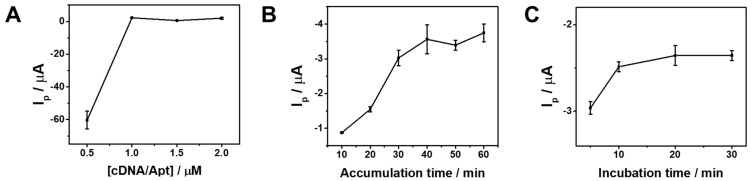
Effect of (**A**) cDNA/Apt concentration in 10 mM Tris-HCl buffer (pH 7.4). (**B**) cDNA/Apt accumulation time for immobilization under the condition of [cDNA/Apt] = 1 µM. (**C**) Incubation time of OTA under the conditions of accumulation = 40 min, [cDNA/Apt] = 1 µM, and [OTA] = 1 nM.

To ensure reliable and reproducible electrochemical measurements, key operational parameters were optimized prior to analytical evaluation. As shown in Figure 4A, the current response increased markedly with increasing cDNA/MB-Apt concentration up to 1.0 μM and subsequently reached a plateau, indicating saturation of available absorbing sites on the electrode surface. Accordingly, 1.0 μM was selected as the optimal probe concentration. The influence of accumulation time was further examined (Figure 4B). The current response gradually increased with prolonged accumulation time and stabilized after approximately 40 min, suggesting that adsorption equilibrium had been established at the electrode interface. Therefore, 40 min was chosen as the optimal accumulation time. Similarly, the effect of OTA incubation time was investigated (Figure 4C). The current response increased with increasing incubation time and reached a plateau at 20 min, indicating sufficient target binding and completion of the aptamer conformational transition. Thus, an incubation time of 20 min was selected for subsequent electrochemical sensing measurements.

### 3.3. Electrochemical Sensing Performance

**Figure 5:**
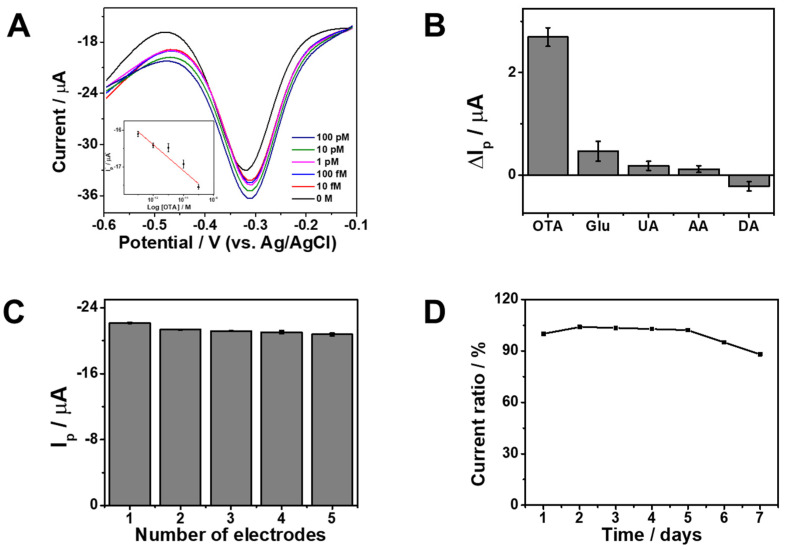
(**A**) SWV responses of the cDNA/Apt/ERGO-GCE toward various OTA concentrations of 0 M to 100 pM in 10 mM Tris-HCl buffer (pH 7.4). Inset: Corresponding calibration curve between [OTA] and cathodic reduction peak (I_p_). (**B**) Selectivity of the sensors over other interferents. (**C**) Reproducibility of the sensors for five different modified electrodes with triplicate measurements (*n* = 3). (**D**) Stability of the sensor in 7 days in 10 mM Tris-HCl buffer (pH 7.4).

The analytical performance of the proposed aptasensor was systematically evaluated by monitoring the square-wave voltammetry (SWV) response to increasing concentrations of OTA under optimized conditions. As shown in Figure 5A, the peak current increased progressively with increasing OTA concentration, demonstrating a concentration-dependent response consistent with a turn-on sensing mechanism. A linear relationship was obtained between I_p_ and log[OTA] from 10 fM to 100 pM (R^2^ = 0.94). The slight deviation at the lowest concentrations is attributed to the inherently low signal level near the detection limit; nevertheless, the calibration maintained reliable quantitative performance within the tested range. The limit of detection (LOD), calculated using the 3σ/s criterion (where σ represents the standard deviation of the blank signal and s is the slope of the calibration curve), was determined to be 0.67 fM, indicating high sensitivity. The observed analytical performance can be attributed to the specific recognition between OTA and its corresponding aptamer, together with the favorable electrical conductivity and increased electroactive surface area provided by the ERGO interface. These factors collectively facilitate efficient electron transfer and enhance the electrochemical signal output. A comparison with previously reported electrochemical OTA sensors (Table 1) confirms that the present platform exhibits competitive sensitivity within the reported range. All experiments were conducted in triplicate (*n* = 3), and the results are presented as mean ± standard deviation. The selectivity of the sensor was further assessed in the presence of common interfering species, including glucose (Glu), uric acid (UA), ascorbic acid (AA), and dopamine (DA). As illustrated in Figure 5B, OTA produced a significantly larger change in peak current (ΔI_p_) compared to the minimal responses observed for the interfering analytes, confirming the specific binding capability of the aptamer toward OTA under the experimental conditions. Reproducibility was evaluated using five independently fabricated electrodes under identical experimental conditions (Figure 5C). The resulting current responses showed low variation, indicating reliable fabrication consistency. Stability was examined by storing the sensor at 4 °C in Tris–HCl buffer for 7 days (Figure 5D). The sensor retained a high proportion of its initial response, demonstrating acceptable short-term storage stability. Overall, the ERGO based aptasensor enables sensitive and selective detection of OTA, with satisfactory reproducibility and operational stability under the tested conditions.

## 4. Conclusions

In this study, an electrochemical turn-on aptasensor for the detection of ochratoxin A (OTA) was developed using an electrochemically reduced graphene oxide (ERGO)-modified electrode. Material and electrochemical analyses verified the successful reduction in graphene oxide and the stepwise construction of the cDNA/MB-Apt sensing interface. The proposed sensor exhibited a linear response over the concentration range of 10 fM to 100 pM, with a limit of detection of 0.67 fM. In addition, the platform demonstrated satisfactory selectivity against common interfering species, as well as acceptable reproducibility and short-term storage stability. The analytical performance can be explained by the combined effect of the ERGO substrate, which enhances electron transfer and provides an increased electroactive surface area, and the specific binding interaction between OTA and its aptamer. Overall, the developed sensing strategy offers a feasible approach for sensitive OTA detection and may serve as a basis for further development of electrochemical aptamer-based sensing systems for food and environmental analysis.

## Figures and Tables

**Table 1:** Performance comparison between the present work and previously reported OTA sensors.

Method	Linear Range (M)	LOD (M)	Reference
Fluorescence (ratiometric)	1.24 × 10^−8^–2.48 × 10^−7^	1.15 × 10^−8^	[53]
Electrochemical immunosensor	1.24 × 10^−10^–4.95 × 10^−7^	4.95 × 10^−13^	[54]
Turn-on fluorescence	0–1.0 × 10^−5^	1.35 × 10^−8^	[55]
Dual-mode (fluorescence–colorimetric) immunosensor	2.48 × 10^−12^–2.48 × 10^−8^	1.29 × 10^−12^	[56]
Fluorescent sensor	1.0 × 10^−8^–4.0 × 10^−7^	9.9 × 10^−10^	[57]
Aptasensor	1.0 × 10^−14^–1.0 × 10^−10^	6.7 × 10^−16^	This work

## Data Availability

The data presented in this study are available on request from the corresponding author.

## Supplementary Materials

The following supporting information can be downloaded at: https://www.mdpi.com/article/10.3390/s26061937/s1, Figure S1: AFM images of (A) bare GCE, (B) ERGO-modified GCE (ERGO-GCE), and (C) cDNA/MB-Apt functionalized ERGO-GCE. Scale bar: 200 nm; Figure S2: Calibration curves for OTA detection. (A) Non-linear response curve showing the dependence of the electrochemical signal on OTA concentration. (B) Linear calibration plot derived from the linear response range used for the calculation of the limit of detection (LOD).
