# Supplementary material for: A Target-Displaced Aptamer–cDNA Duplex Strategy on ERGO for Ultrasensitive Turn-On Electrochemical Detection of Ochratoxin A
Source: Sensors (Basel). 2026 Mar 19;26(6):1937. doi: 10.3390/s26061937 (PMC13030630; doi:10.3390/s26061937)
Supplement: Supplementary file 1 [file sensors-26-01937-s001.zip › sensors-4189999-supplementary.pdf]

Supplementary material

for

A Target-Displaced Aptamer–cDNA Duplex Strategy on ERGO for Ultrasensitive Turn-On Electrochemical Detection of Ochratoxin A

Intan Gita Lestari†, Seung Joo Jang†, and Tae Hyun Kim *

Department of Chemistry, Soonchunhyang University, Republic of Korea

* Correspondence to. thkim@sch.ac.kr (T. H. Kim)

Tel.: +82-41-530-4722 (T. H. Kim)

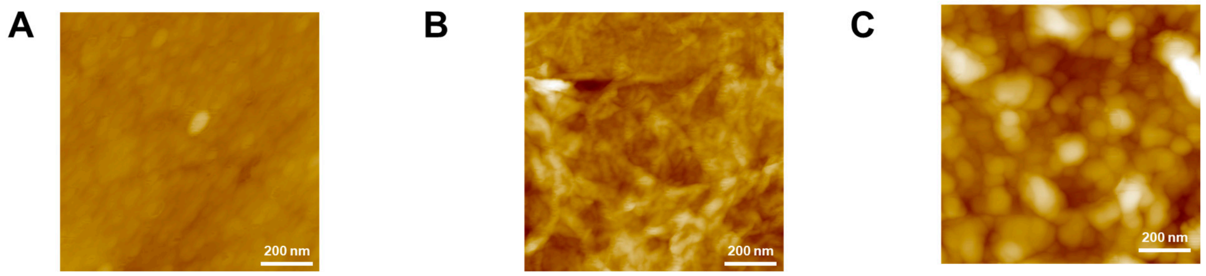

Figure S1. AFM images of (a) bare GCE, (b) ERGO-modified GCE (ERGO-GCE), and (c) cDNA/MB-Apt functionalized ERGO-GCE. Scale bar: 200 nm.

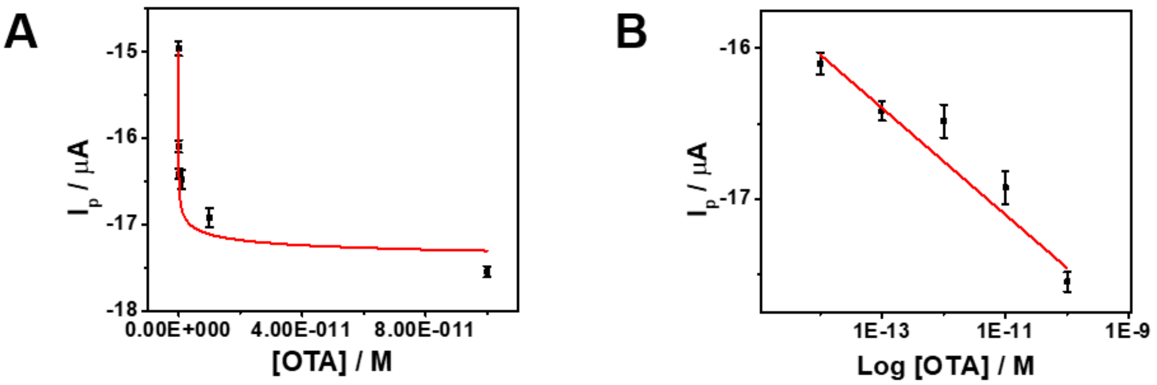

Figure S2. Calibration curves for OTA detection. (a) Non-linear response curve showing the dependence of the electrochemical signal on OTA concentration. (b) Linear calibration plot derived from the linear response range used for the calculation of the limit of detection (LOD).
